# Effects of Long-Term Environmental Enrichment on Anxiety, Memory, Hippocampal Plasticity and Overall Brain Gene Expression in C57BL6 Mice

**DOI:** 10.3389/fnmol.2016.00062

**Published:** 2016-08-03

**Authors:** Melanie Hüttenrauch, Gabriela Salinas, Oliver Wirths

**Affiliations:** ^1^Division of Molecular Psychiatry, Department of Psychiatry and Psychotherapy, University Medical Center, Georg-August-UniversityGöttingen, Germany; ^2^Department of Developmental Biochemistry, DNA Microarray and Deep-Sequencing Facility, University Medical CenterGöttingen, Germany

**Keywords:** enriched environment, Morris water maze, dentate gyrus, deep sequencing, neurogenesis, physical activity, stereology, hippocampal plasticity

## Abstract

There is ample evidence that physical activity exerts positive effects on a variety of brain functions by facilitating neuroprotective processes and influencing neuroplasticity. Accordingly, numerous studies have shown that continuous exercise can successfully diminish or prevent the pathology of neurodegenerative diseases such as Alzheimer’s disease in transgenic mouse models. However, the long-term effect of physical activity on brain health of aging wild-type (WT) mice has not yet been studied in detail. Here, we show that prolonged physical and cognitive stimulation, mediated by an enriched environment (EE) paradigm for a duration of 11 months, leads to reduced anxiety and improved spatial reference memory in C57BL6 WT mice. While the number of CA1 pyramidal neurons remained unchanged between standard housed (SH) and EE mice, the number of dentate gyrus (DG) neurons, as well as the CA1 and DG volume were significantly increased in EE mice. A whole-brain deep sequencing transcriptome analysis, carried out to better understand the molecular mechanisms underlying the observed effects, revealed an up-regulation of a variety of genes upon EE, mainly associated with synaptic plasticity and transcription regulation. The present findings corroborate the impact of continuous physical activity as a potential prospective route in the prevention of age-related cognitive decline and neurodegenerative disorders.

## Introduction

Given the fact that no effective drugs to treat dementia disorders are available to date, the research focus has more and more shifted toward more promising preventive approaches in recent years. In the last decade, physical inactivity has been repeatedly shown to be one of the major risk factors associated with dementia (Barnes and Yaffe, 2011; Norton et al., 2014). A study by Weuve et al. (2004) was one of the first to show that long-term physical activity enhances cognitive function and lessens cognitive decline in older women (Weuve et al., 2004). Furthermore, a recently published 29-years-long follow-up study with a Finnish twin cohort disclosed that long-term vigorous physical activity during adulthood significantly decreases the risk to develop dementia in later life and leads to a lower mortality rate (Iso-Markku et al., 2015). Importantly, beneficial effects of regular exercise have also been reported in subjects already suffering from mild cognitive impairment (MCI) or dementia (Heyn et al., 2004; Nagamatsu et al., 2013).

Proposed mechanisms disclosing how physical activity lowers the risk of dementia in humans are diverse and include enhanced cerebral blood flow as well as synthesis of neurotransmitters among others (Coelho et al., 2014). Furthermore, it has been reported that higher levels of aerobic exercise are associated with an increased hippocampal volume and improved spatial memory in older subjects (Erickson et al., 2011) and that physical activity reduces hippocampal atrophy in individuals at genetic risk for AD (Smith et al., 2014).

The positive effects of regular exercise on brain health in humans has been tried to be explained by data obtained in experimental animal models. Therefore, rodents are subjected to environmental enrichment (EE) paradigms, which mimic a cognitively and physically active lifestyle (Nithianantharajah and Hannan, 2006). A large amount of recent studies have reported that, e.g., rodent AD models maintained under enriched living conditions show improved performances in hippocampus-dependent cognitive tests such as the Morris water maze (MWM; Polito et al., 2014; Rao et al., 2015; Huttenrauch et al., 2016). Consistent with improved spatial memory performance, exposure to physically stimulating environments has been shown to induce structural and functional changes in various brain regions, with the hippocampus being specifically sensitive to exercise (Cotman and Berchtold, 2007). The effects of physical activity may be partially mediated through the induction of growth factors such as brain derived neurotrophic factor (BDNF), insulin-like growth factor (IGF; Gomez-Pinilla et al., 2002; Cassilhas et al., 2012) or effects on hippocampal neurogenesis (Fabel et al., 2009; Garthe et al., 2016), however, the precise molecular mechanisms through which exercise benefits the brain remain to be elucidated. The ultimate goal would be the development of “enviromimetic” drugs, which mimic or increase the effect on the brain induced by environmental enrichment. Therefore, we aimed on further investigating the molecular changes upon physical activity by performing a transcriptome analysis using a deep sequencing approach in C57BL6 wild-type (WT) mice subjected to prolonged environmental enrichment. Here, we investigated the long-term effect of environmental enrichment lasting from weaning until 12 months of age in C57Bl6/J mice. We demonstrate that continuous mental and physical stimulation improves long-term spatial reference memory and increases the number of neurons in the dentate gyrus (DG). Finally, a deep sequencing analysis identified novel genes that may mediate the beneficial effects of prolonged periods of exercise.

## Materials and Methods

### Mice and Housing conditions

**FIGURE 1:**
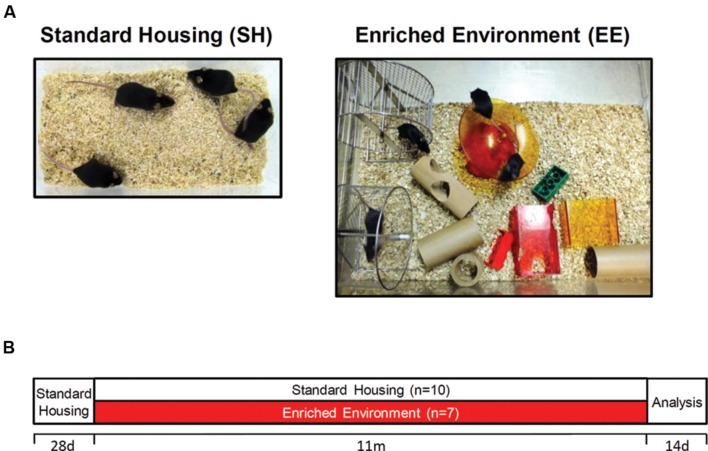
**Housing conditions and experimental design.** **(A)** Exemplary pictures of standard (SH) and enriched (EE) housing conditions. Mice were housed in groups of 3–5. Enriched cages were equipped with three running wheels, tunnels, houses, and toys. **(B)** After weaning, C57Bl6/J mice were randomly allocated to either SH or EE conditions for 11 months. With 12 months of age, mice were tested in the open field and Morris water maze test followed by sacrifice and tissue collection.

With 1 month (m) of age, female C57Bl6/J mice were randomly distributed to either standard housing (SH; *n* = 10) or enriched housing (EE; *n* = 7) conditions until the age of 12 months. For SH conditions, standard laboratory cages were used (33 cm × 18 cm × 14 cm), whereas mice maintained under EE conditions were housed in larger rat cages (55 cm × 34 cm × 20 cm). EE cages were equipped with three running wheels, nesting material, tunnels, shelters, houses and toys, which were changed and rearranged weekly to increase the sense of novelty (**Figure 1**). Mice were housed in groups of 3–5 to ensure social interactions. In all conditions, food and water were provided *ad libitum*. Only female mice were used in the present study, as it has been shown that long-term enriched housing leads to enhanced aggressive behavior of male mice (Marashi et al., 2003). All animals were handled according to the German guidelines for animal care and experiments have been approved by the local animal care and use committee [Landesamt für Verbraucherschutz und Lebensmittelsicherheit (LAVES), Lower Saxony].

### Behavioral Tasks

#### Open Field

The open field test was used to assess both exploratory behavior and locomotor activity. The mice were tested using an open field box made of gray plastic with a 50 cm × 50 cm surface area and 38 cm-high walls. Monitoring was done by an automated tracking system (ANY-Maze, Stoelting). The behavioral parameters registered during 5-min sessions were (1) the percentage of time spent in the central part (20 cm × 20 cm) versus total time, (2) total traveled distance, (3) average speed, and (4) total time of activity.

#### Morris Water Maze

The MWM was carried out as described previously (Bouter et al., 2014). In brief, mice were initially subjected to 3 days of cued training, during which a triangular flag marked the platform position. Between different trials (*n* = 4 per day), both the location of the platform and the position where mice were introduced into the pool was changed. Twenty-four hours after the last trial of the cued training, mice performed another 5 days of acquisition training, in which the flag was removed from the platform. In addition to distal cues in the testing room, proximal visual cues were attached to the outside of the pool. Throughout the training, the platform location remained stationary for each mouse, while trials were conducted as during the cued training phase. A probe trial used to assess spatial reference memory was performed 24 h after the last acquisition trial. In this test, the platform was removed from the pool, and mice were introduced from a novel entry point. Mice were then allowed to swim freely for 1 min while their swimming path was recorded using an automated video tracking system (ANY-Maze, Stoelting).

#### Quantification of Neuron Numbers Using Unbiased Stereology

Mice were transcardially perfused with phosphate-buffered saline (PBS) and brains were carefully dissected. The left hemisphere was fixed in 4% paraformaldehyde, cryoprotected in 30% sucrose and deep-frozen; the right hemisphere was immediately snap-frozen and stored at -80°C for biochemical analysis. Stereological analysis of the hippocampal cell layer CA1 (Bregma -1.22 to -3.80 mm) and the DG (Bregma -1.34 to -3.80 mm) using a stereology workstation (Olympus BX51 with a motorized specimen stage for automatic sampling and a 100x oil lens (numerical aperture [NA] = 1.35)), StereoInvestigator 7 (MicroBrightField, Williston, VT, USA) were performed as previously described on cresyl-violet stained sections (Cotel et al., 2012).

#### Analysis of Neurogenesis

A series of every 10th coronal frozen section of 30 μm thickness was processed in a free-floating staining protocol to quantify the number of newborn neurons. Briefly, sections were rehydrated in PBS and endogenous peroxidase activity was blocked by incubation in PBS including hydrogen peroxide for 20 min. After washing in PBS including Triton x-100, unspecific antibody binding was blocked by incubation in PBS including 10% fetal calf serum (FCS) and 4% skim milk. The primary antibody against doublecortin (DCX, 1: 200, Santa Cruz Biotechnology, Santa Cruz, CA, USA) was incubated overnight, followed by incubation with a secondary biotinylated antibody. Staining was visualized using the ABC method using a Vectastain kit (Vector Laboratories) and DAB as chromogen. The overall number of newborn neurons was counted in the subgranular zone (SGZ) of the DG using the meander scan option of StereoInvestigator to quantify all DCX-positive cells in a given section. The resulting neuron number was multiplied by 10 to obtain the total number of newborn neurons (Cotel et al., 2012).

#### Deep Sequencing Analysis

Total RNA was prepared from WT SH and EE brain hemispheres (*n* = 6 each) using Trifast reagent (Peqlab) according to the protocol of the supplier. As starting material for the library preparation, 0.5 μg of total RNA was used. The libraries were generated according to the TruSeq mRNA Sample Preparation Kits v2 Kit from Illumina (Cat. N°RS-122-2002). The fluorometric based QuantiFluor^TM^ dsDNA System from Promega (Mannheim, Germany) was used for accurate quantitation of cDNA libraries. The size of final cDNA libraries was determined by using the Fragment Analyzer from Advanced Bioanalytical. cDNA libraries were amplified and sequenced by using the cBot and HiSeq2000 from Illumina (SR; 1 × 50 bp; ca. 30 Mio reads per sample). Sequence images were transformed to bcl files using Illumina software BaseCaller, which were demultiplexed to fastq files with CASAVA v1.8.2 and quality checks were done via fastqc.

Read alignment was performed using STAR v2.3.0 to the hg19 reference genome, while data conversion and sorting was carried out with samtools 0.1.19^1^ and reads per gene were counted via htseq version 0.6.1^2^. Data analysis was performed using R/Bioconductor (3.0.2/2.12^3^) with DESeq2 and gplots packages. Candidate genes were filtered to a minimum of FDR-corrected *p*-value < 0.05 as described previously (Huttenrauch et al., 2016).

#### Quantitative Real-Time Polymerase Chain Reaction (RT-PCR)

Real-time reverse transcription-polymerase chain reaction (RT-PCR) was used to confirm deep sequencing results for a subset of genes from those fulfilling the criteria of significant expression differences (*p* < 0.05) on a random selection basis. Deep frozen brain hemispheres from WT SH and EE (*n* = 6 each) were homogenized in TriFast reagent (Peqlab) essentially as previously described (Huttenrauch et al., 2015). Primer sequences are described in the supplementary information and relative expression levels were calculated using the 2^-ΔΔCt^ method (Livak and Schmittgen, 2001) and normalized to housekeeping gene β-Actin. The expression ratio results of the studied transcripts are tested for significance by unpaired *t*-test. Levels of significance were labeled as follows: ^∗∗∗^*p* < 0.001; ^∗∗^*p* < 0.01; ^∗^*p* < 0.05.

## Results

**FIGURE 2:**
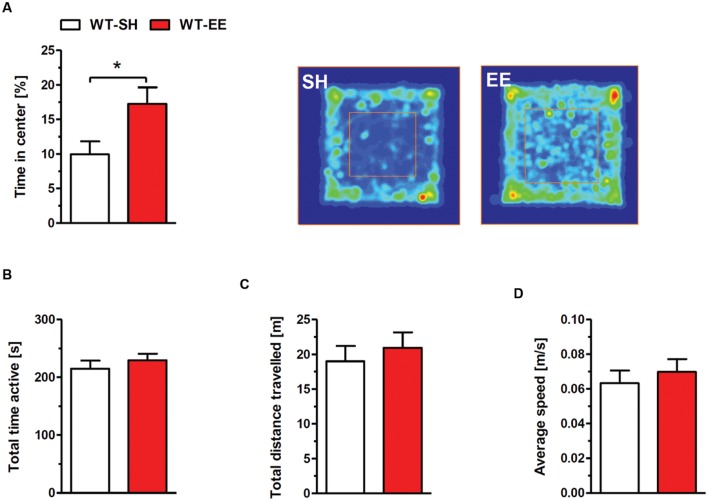
**Reduced anxiety upon enriched living conditions.** **(A)** Enriched housed WT mice spent significantly more time in the center region of the open field compared to sedentary controls. The occupancy plots indicate exemplarily the running traces of SH and EE mice during the 5-min open field session. However, there was no difference in total time active **(B)**, total distance traveled **(C)** and average speed **(D)** between standard and enriched C57Bl6/J mice. Unpaired *t*-test. ^∗^*p* < 0.05. All data were given as mean ± SEM (*n* = 7–10 per group).

The exploratory and spontaneous locomotor activity of enriched housed WT mice was compared to standard controls in the open field paradigm. No significant differences in the total time of activity (**Figure 2B**), the total distance traveled (**Figure 2C**) and the average speed (**Figure 2D**) were detected between the two groups. The time spent in the center region can be used as a measure of anxiety-related behavior. Mice maintained under EE conditions spent significantly more time in the center region when compared to sedentary controls (*p* < 0.05; **Figure 2A**), indicating a loss of anxiety.

**FIGURE 3:**
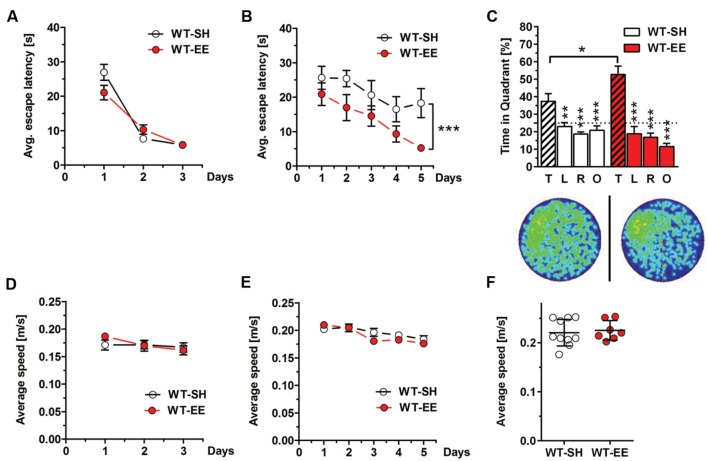
**Improved spatial memory performance in WT mice upon EE housing.** **(A)** WT-SH and EE mice showed progressively decreasing escape latencies over the 3 days of cued training. **(B)** Similar to the cued training, SH- and EE-WT mice showed progressively reduced escape latencies over the 5 days of acquisition training. However, WT-EE mice displayed an improved spatial learning performance compared to their sedentary controls as seen by lower escape latencies over the whole training period. **(C)** WT-SH and EE mice displayed an intact spatial reference memory as they showed a clear and significant preference for the target (T) compared to all the other quadrants (L, R, O), with EE mice spending significantly more time in the target quadrant when compared to SH mice. The occupancy plots indicate the averaged swimming traces of standard and enriched housed mice during the probe trial. **(D–F)** No differences in swimming speed were observed between WT-SH and EE mice in cued training, acquisition training and probe trial. **(A,B,D,E)** Two-way repeated measures ANOVA. **(C)** One-way ANOVA followed by Bonferroni multiple comparisons. **(F)** Unpaired *t*-test. ^∗∗∗^*p* < 0.001; ^∗∗^*p* < 0.01; ^∗^*p* < 0.05. All data were given as mean ± SEM (*n* = 7–10 per group. L, left; R, right; O, opposite).

Spatial reference memory was assessed in standard and enriched housed WT mice using the MWM task. 12-month-old WT mice housed under SH and EE conditions showed progressively decreased escape latencies over 3 days of cued training as expected (**Figure 3A**), while no differences in swimming speed were detected (**Figure 3D**). During the following acquisition training phase, mice housed under enriched conditions showed significantly reduced escape latencies compared to their sedentary controls (Two-way repeated measures ANOVA, *p* = 0.0187; **Figure 3B**), indicating improved spatial learning. Again, no differences in swimming speed were detected (**Figure 3E**). During the final probe trial, both WT SH and EE mice showed a clear preference for the target quadrant, indicating that both groups learned the task. However, mice housed under EE conditions showed a significantly increased time in the target quadrant compared to the sedentary controls (*t*-test, *p* = 0.0354; **Figure 3C**), with again unchanged swimming speed (**Figure 3F**). Representative occupancy plots confirmed that enriched-housed WT mice focussed their search on the initial platform position, while sedentary mice showed a more random search profile (**Figure 3C**).

**FIGURE 4:**
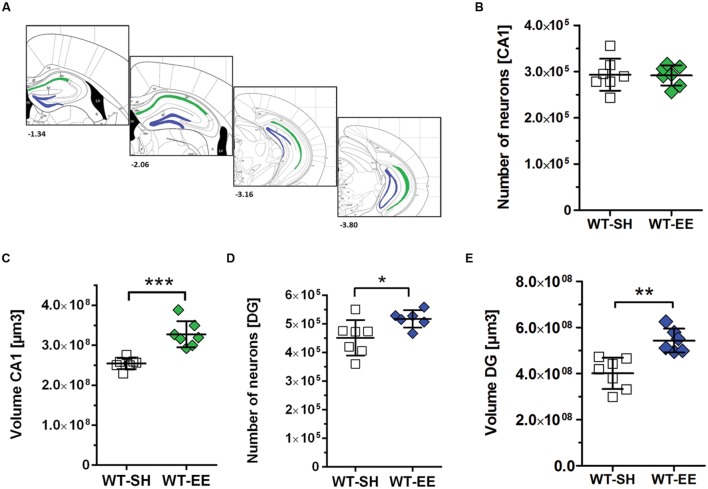
**The effect of prolonged enrichment on hippocampal neuron numbers and hippocampal volume in WT mice.** **(A)** Schematic representation of hippocampal counting areas. The CA1 region (outlined in green) was counted from Bregma -1.22 mm to -3.80 mm. The granule cell layer of the dentate gyrus (outlined in blue) was counted from Bregma -1.34 mm to -3.80 mm (modified from Paxinos and Franklin, 2001). **(B)** Design-based stereological analysis revealed no difference in CA1 neuron numbers between SH and EE mice. **(C)** The CA1 volume was significantly increased in WT mice housed under enriched living conditions when compared to sedentary controls (+29%). **(D)** Stereological analysis revealed a 15% increased dentate gyrus neuron number in EE compared to SH mice. **(E)** Analysis of dentate gyrus volume revealed a 36% increase in EE compared to SH mice. **(B–E)** Unpaired *t*-test. ^∗∗∗^*p* < 0.001; ^∗∗^*p* < 0.01; ^∗^*p* < 0.05. All data were given as mean ± standard deviation (SD); (*n* = 6–7 per group).

**FIGURE 5:**
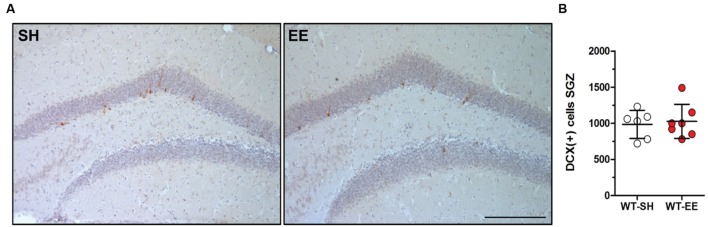
**Enriched environment had no effect on adult neurogenesis in 12-month-old WT mice.** **(A,B)** New-born doublecortin (DCX)-positive neurons were stained and quantified in the subgranular zone (SGZ) of the dentate gyrus. Adult neurogenesis was found to be unchanged upon long-term enriched living conditions in C57Bl6/J mice. Unpaired *t*-test. All data were given as mean ± standard deviation (SD); (*n* = 7 per group). Scale bar: 100 μm.

In order to evaluate whether the improved spatial reference memory performance can be attributed to an altered hippocampal neuron number, stereological quantifications of CA1 and DG neuron numbers were performed (**Figure 4A**). Between SH and EE housed WT mice, no significant difference in the number of CA1 neurons could be detected (WT-SH: 293319 ± 34662; WT-EE: 291751 ± 21897; *p* > 0.05; **Figure 4B**). A quantitative analysis of the CA1 volume instead revealed a significant increase of ∼29% in EE compared to SH animals (*p* = 0.0001; **Figure 4C**). We next analyzed whether an altered number of DG neurons might contribute to the observed beneficial effects of the EE paradigm. Mice housed under EE conditions harbored ∼15% more DG neurons than sedentary mice (WT-SH: 451001 ± 61864; WT-EE: 517122 ± 30443; *p* < 0.05; **Figure 4D**). An analysis of the DG volume also revealed an increase of ∼36% in EE compared to SH mice (*p* = 0.0015; **Figure 4E**). In order to evaluate, if an increase in the neurogenesis rate contributed to the altered DG neuron number, DCX-positive neurons were quantified in the SGZ in both EE and sedentary WT mice. No significant differences were detected between WT-SH (985.0 ± 195.4) and WT-EE mice (1027.0 ± 236.2; **Figure 5**).

**FIGURE 6:**
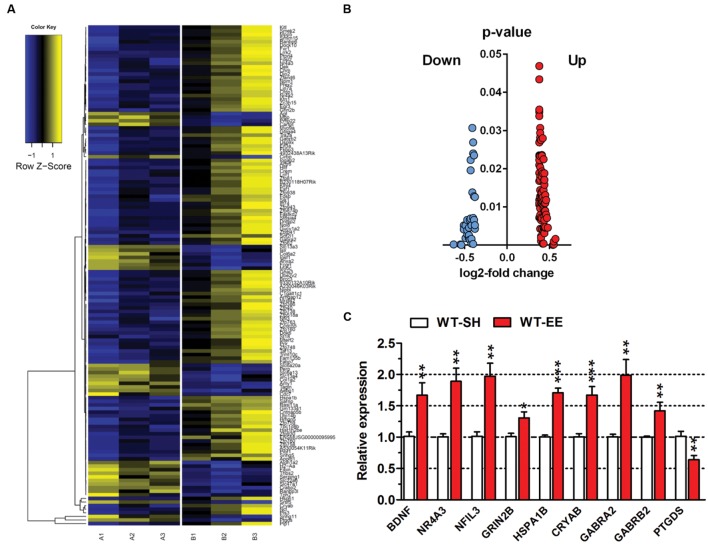
**Gene expression profile changes upon long-term EE in C57Bl6/J mice.** **(A)** Heatmap of differentially expressed genes (DEGs) between SH and EE WT mice. Each column represents a pooled sample of two brain hemispheres (A1-3 = SH, B1-3 = EE) and each row represents the top genes that were differentially expressed. Yellow indicates upregulated genes, blue indicates downregulated genes. **(B)** Volcano plot of showing up- (red) or down-regulated (blue) genes in 12-month-old WT animals upon long-term enriched housing. **(C)** Deep sequencing data were validated using qRT-PCR analysis. Normalization was performed against the house-keeping gene actin. Unpaired *t*-test. ^∗∗∗^*p* < 0.001; ^∗∗^*p* < 0.01; ^∗^*p* < 0.05. All data were given as mean ± SEM (*n* = 6 per group).

In order to analyze if long-term housing under EE conditions has an influence on the brain gene expression profile of WT mice, a deep sequencing analysis on whole brain hemispheres was performed (**Figure 6A**). Prolonged exposure to enriched conditions led to statistically significant changes in the expression of 137 genes with a log2-fold value of at least 0.38 (corresponding to a change in gene expression of 1.3-fold; Top15 up- or down-regulated genes, **Table 1**). 103 genes were up-regulated and 34 genes were down-regulated in WT EE mice compared to SH littermate controls (**Supplementary Table S1**; **Figure 6B**). A gene ontology analysis of the up-regulated genes using the Database for Annotation, Visualization and Integrated Discovery (DAVID v6.7) bioinformatics package (Huang et al., 2009) revealed significant associations, while only gene ontology categories presenting both *p*-value < 0.05 and Benjamini adjustment < 0.05 were considered: for the up-regulated genes, an association with the GO terms “transcription” (GO:0006351; Benjamini *p*-value 1.8E-3), “regulation of transcription” (GO:0006355; Benjamini *p*-value 4.6E-3), “regulation of RNA metabolic processes” (GO:0051252; Benjamini *p*-value 6.0E-3) were found, whereas down-regulated genes were associated with GO term “extracellular region” (GO:0005576; Benjamini *p*-value 2.3E-5) and “extracellular region part” (GO:0044421; Benjamini *p*-value 2.3E-3).

**Table 1:** Top 15 up- or down-regulated genes in WT-EE compared to WT-SH mice.

ID	Gene name	Gene description	log2 fold change	Adjusted *p*-value
MGI:2385044	Zfp758	Zinc finger protein 758	0.57	1.64E-03
MGI:99517	Hspa1b	Heat shock protein 1B	0.55	9.78E-04
MGI:102858	Fosl2	Fos-like antigen 2	0.55	1.72E-04
MGI:1352457	Nr4a3	Nuclear receptor subfamily 4, group A, member 3	0.54	5.29E-05
MGI:95614	Gabra2	Gamma-aminobutyric acid (GABA) A receptor, subunit alpha 2	0.48	4.56E-03
MGI:95575	Fosb	FBJ osteosarcoma oncogene B	0.47	7.09E-03
MGI:2441787	A230046K03Rik	Riken cDNA A230046K03 gene	0.46	7.98E-03
MGI:2446280	Zfp759	Zinc finger protein 759	0.46	1.18E-02
MGI:3639495	Dok6	Docking protein 6	0.45	8.99E-03
MGI:109495	Nfil3	Nuclear factor, interleukin 3, regulated	0.45	4.73E-03
MGI:1915420	B230118H07Rik	RIKEN cDNA B230118H07 gene	0.45	8.42E-03
MGI:96974	Kitl	Kit ligand	0.45	4.56E-03
MGI:1923173	Dnttip2	Deoxynucleotidyltransferase, terminal, interacting protein 2	0.45	1.10E-02
MGI:2445092	Utp14b	UTP14, U3 small nucleolar Ribonucleoprotein, homolog B (yeast)	0.45	1.31E-02
MGI:1919922	Zfp518a	Zinc finger protein 518A	0.44	6.55E-03
MGI:892001	Slc22a6	Solute carrier family 22 (organic anion transporter), member 6	-0.63	1.72E-04
MGI:106012	Tagln	Transgelin	-0.55	1.87E-04
MGI:98287	Srsf5	Serine/arginine-rich splicing factor 5	-0.54	3.75E -05
MGI:99261	Ptgds	Prostaglandin D2 synthase (brain)	-0.54	9.64E-07
MGI:1914723	Slc47a1	Solute carrier family 47, member 1	-0.51	5.18E-03
MGI:88491	Crabp2	Cellular retinoic acid binding protein II	-0.51	4.13E-03
MGI:2143217	Slc6a20a	Solute carrier family 6 (neurotransmitter transporter), member 20A	-0.49	1.83E-03
MGI:894696	Serping1	Serine (or cysteine) peptidase inhibitor, clade G, member 1	-0.49	5.13E-03
MGI:88468	Col1a2	Collagen, type I, alpha 2	-0.47	6.61E-03
MGI:88019	Amy1	Amylase 1, salivary	-0.47	2.77E-03
MGI:1197012	Aebp1	AE binding protein 1	-0.45	3.88E-03
MGI:98738	Thbs2	Thrombospondin 2	-0.45	4.56E-03
MGI:2147913	Igsf1	Immunoglobulin superfamily, member 1	-0.45	1.83E-03
MGI:88246	Anxa2	Annexin A2	-0.45	2.58E-03
MGI:87963	Agt	Angiotensinogen (serpin peptidase inhibitor, clade A, member 8)	-0.44	1.40E-03


To validate the differentially expressed genes (DEGs) identified by deep sequencing analysis, both up- and downregulated candidate genes were randomly selected and verified by qRT-PCR. For all selected genes, the qRT-PCR analysis confirmed the expression levels of the deep sequencing results (**Figure 6C**).

## Discussion

In recent years, great progress has been made in investigating the effect of physical activity on disease progression in preclinical models of Alzheimer’s disease and other neurodegenerative disorders by the use of environmental enrichment paradigms. However, cognitive impairment, dysregulation of neurogenesis and brain atrophy are not only associated with diseases, but also with normal aging. Therefore, the aim of the present study was to elucidate, whether a prolonged environmental enrichment paradigm, providing both cognitive stimulation and increased physical activity (Fabel et al., 2009), has an impact on behavioral performance, hippocampal neuron numbers and cerebral gene expression changes in aging WT mice. The enrichment was started after weaning at the age of 4 weeks and was continued until the age of 12 months. Increased stress levels have been associated with enhanced anxiety phenotypes. Although corticosterone levels have not been assessed in the present study, it has been previously shown that beneficial effects of a 2-months exercise (Cassilhas et al., 2012) or 6-months enrichment period (Cracchiolo et al., 2007) occurred without any changes in blood cytokine or corticosterone levels. Animals allowed to exercise showed a significantly increased time in the center of the open field task, corresponding to a reduced anxiety phenotype and confirming previous data (Benaroya-Milshtein et al., 2004; Kazlauckas et al., 2011). Therefore, we do not expect that altered stress levels affect the behavioral analysis in the present study. Analysis of long-term spatial reference memory using the MWM task revealed a significantly increased performance in the probe trial of enriched compared to sedentary mice. This finding corroborates previous reports describing an improved spatial memory task performance following long-term running in rodent AD models (Garcia-Mesa et al., 2011; Huttenrauch et al., 2016), while related results have been also obtained in WT mice using shorter enrichment protocols (Garthe et al., 2016). However, most available reports use enrichment protocols with much shorter duration of mainly up to 6 months. It has been previously demonstrated that enriched housing increases DG neurogenesis (van Praag et al., 1999; Kobilo et al., 2011), even in aged mice (Kempermann et al., 2002) and that hippocampal neurogenesis is a mechanism critically involved in learning processes (Garthe et al., 2016). On the other hand, strongly decreased overall neurogenesis rates during aging have been also reported (Kuhn et al., 1996; Merkley et al., 2014). In good agreement, the number of new-born doublecortin (DCX)-positive neurons was unaltered between enriched and sedentary WT mice, however, stereological analysis revealed a significantly increased total neuron number in the DG in WT EE, which might reflect an increase in neurogenesis and survival of new born cells in earlier life periods. WT mice maintained under a cognitively and physically stimulating environment displayed a larger hippocampal volume, which suggests that improved cognitive performances are correlated to structural changes in the brain. This is in good agreement with human studies showing that physical activity is associated with an increased volume of the hippocampus (Erickson et al., 2011). Enrichment-induced increases in the CA1 spine density in mice (Rampon et al., 2000), as well as enhanced dendritic arborization and increased dendritic spine density in layer III cortical neurons (Leggio et al., 2005) have been previously reported. In accordance with our finding, significant increases in the CA1 volume, as well as CA1 and DG dendrite length have been demonstrated in enriched mice using Golgi-Cox staining (Faherty et al., 2003). Interestingly, increased numbers of CNPase-positive oligodendrocytes have been detected in CA1 and DG of enriched rats (Zhao et al., 2013) which might also contribute to an overall volume increase in these brain regions. Very recently, chronic imaging of pyramidal cell morphology revealed that exposure to EE results in a 30–40% increase in spine density in 3–4 month-old animals. This could be confirmed in adult animals to a slightly lesser degree (20–30%; Jung and Herms, 2014).

An up-regulation of the gene encoding BDNF has been repeatedly reported (Gomez-Pinilla et al., 2002; Adlard et al., 2005) and is believed to act as a mediator of the efficacy of exercise on synaptic plasticity and cognition (Vaynman et al., 2004). Therefore BDNF served as a control measure in the current study and the *BDNF* mRNA levels were found to be significantly up-regulated following 11 months of enrichment.

The objective of the transcriptome analysis using deep sequencing was to identify novel genes that might play a role in mediating the beneficial effects of long-term physical activity and environmental enrichment. Changes in anxiety-related behaviors, as reflected in the open field analysis, appear to be regulated by an interconnected system of brain structures including the basolateral amygdala and as lesions in a variety of brain regions including hippocampus, striatum, but also cerebellum and cerebral cortex were shown to impair MWM performance (D’Hooge and De Deyn, 2001), a whole brain RNA-sequencing approach was performed. On the other hand, this approach also includes limitations, as global gene-expression changes cannot be directly related to the morphological alterations detected in the hippocampus and enrichment-specific differences in hippocampal gene expression might be covered or thinned out.

The gene *PTGDS* encoding prostaglandin D2 synthase was one of the most strongly down-regulated genes that could be identified. It is expressed mainly in astrocytes and oligodendrocytes and acts as a neuromodulator (Urade and Hayaishi, 2000). *In vitro* experiments have demonstrated that prostaglandin D2 mediates neuronal damage by mimicking the effects of Aβ1-42, leading to microglial activation and neuron loss (Bate et al., 2006). *PTGDS* has been also identified to be down-regulated in the senescence-accelerated SAMP8 mouse model (Alvarez-Lopez et al., 2013), as well as in the Tg4-42 mouse model of AD, where long-term physical activity led to an amelioration of hippocampal neuron loss and a rescue of behavioral deficits (Huttenrauch et al., 2016).

Several gamma-aminobutyric acid receptor subunits (*GABRA2*, *GABRB2)* were found to be up-regulated in mice maintained under enriched environment (EE) conditions. Further, *GRIN2B* encoding the *N*-methyl D-Aspartate receptor subtype 2B (NMDA2B, NR2B) is up-regulated following long-term exercise, which could be confirmed by RT-PCR analysis. This finding corroborates earlier studies describing increased NR2B levels in the DG of wheel-running Sprague-Dawley rats (Farmer et al., 2004), as well as in the 3xTg mouse model of AD after 6 months of voluntary exercise (Revilla et al., 2014). Expression levels of the NR2B subunit are selectively decreased in the hippocampus and entorhinal cortex of AD patients compared controls (Bi and Sze, 2002) and an up-regulation of NR2B subunit expression is suggested to enhance synaptic plasticity and memory functions in a broad range of behavioral tasks in rodents (Wang et al., 2014).

Information about effects of physical activity on changes of GABAergic neurotransmission are scarce, however, it has been demonstrated that high anxiety rats subjected to chronic restraint stress showed decreased expression of alpha-2 GABA-A subunits in motor cortex and DG (Wisłowska-Stanek et al., 2013). In contrast, EE mice in the current study show reduced anxiety together with increased GABRA2 expression levels. In good agreement, the N900 mouse strain selectively bred for aggression showed an increased anxiety phenotype together with selective reductions in alpha-2 GABA-A subunit levels in frontal cortex and amygdala (Nehrenberg et al., 2009). The reduced anxiety phenotype together with increased GABRA2 expression observed in the present study supports such an association.

Two of the most strongly up-regulated genes were *FOSB* and *FOSL2*. Members of the Fos family can dimerize with proteins of the Jun family to form the transcription factor complex AP-1 (Curran and Franza, 1988). It has been previously demonstrated that the levels of the truncated splice form of FosB, ΔFosB, are potently triggered by long-term physical exercise in the hippocampus and cortex of C57BL/6 mice (Nishijima et al., 2013).

Genes belonging to chaperone families implicated in protein processing within the endoplasmatic reticulum (ER) were also upregulated (*CRYAB*, *HSPA1B*, *HSPH1*). These genes play important roles within ER-associated degradation pathways and are necessary for quality control during protein synthesis by assisting proper folding and protein modifications (Liu and Chang, 2008). HspB5 or α-B-Crystallin (Cryab) has been previously demonstrated to be induced in the brain as a function of short-term exercise in rats (Hu et al., 2009), as well as HSP70 (HspA1B) in the hippocampus and prefrontal cortex following swimming exercise (Liu et al., 2010). We could recently show that a group of similar HSPs were also upregulated upon EE in a novel mouse model for AD, indicating a common mechanism associated with long-term cognitive and physical stimulation (Huttenrauch et al., 2016).

## Conclusion

We provide evidence that prolonged physical activity results in reduced anxiety levels, improved learning behavior and an altered gene expression profile in WT mice. This corroborates findings from previous studies using more short enrichment protocols and supports epidemiological data from human aging and AD obtained in retrospective settings.

## Author Contributions

MH, GS, and OW performed experiments or analyzed data. OW designed the study and wrote the manuscript together with MH. All authors read and approved the final manuscript.

## Conflict of Interest Statement

The authors declare that the research was conducted in the absence of any commercial or financial relationships that could be construed as a potential conflict of interest.

## Footnotes

1 http://www.htslib.org

2 http://www-huber.embl.de/HTSeq

3 http://www.bioconductor.org
