# Supplementary material for: Effects of Long-Term Environmental Enrichment on Anxiety, Memory, Hippocampal Plasticity and Overall Brain Gene Expression in C57BL6 Mice
Source: Front Mol Neurosci. 2016 Aug 3;9:62. doi: 10.3389/fnmol.2016.00062 (PMC4971077; doi:10.3389/fnmol.2016.00062)
Supplement: Supplementary file 1 [file Table_1.DOCX]

| **ID** | **Gene name** | **Gene description** | **log2 Fold change** | **Adjusted p-value** |
| --- | --- | --- | --- | --- |
| MGI:2385044 | Zfp758 | zinc finger protein 758 | 0,57 | 1,64E-03 |
| MGI:99517 | Hspa1b | heat shock protein 1B | 0,55 | 9,78E-04 |
| MGI:102858 | Fosl2 | fos-like antigen 2 | 0,55 | 1,72E-04 |
| MGI:1352457 | Nr4a3 | nuclear receptor subfamily 4, group A, member 3 | 0,54 | 5,29E-05 |
| MGI:95614 | Gabra2 | gamma-aminobutyric acid (GABA) A receptor, subunit alpha 2 | 0,48 | 4,56E-03 |
| MGI:95575 | Fosb | FBJ osteosarcoma oncogene B | 0,47 | 7,09E-03 |
| MGI:2441787 | A230046K03Rik | RIKEN cDNA A230046K03 gene | 0,46 | 7,98E-03 |
| MGI:2446280 | Zfp759 | zinc finger protein 759 | 0,46 | 1,18E-02 |
| MGI:3639495 | Dok6 | docking protein 6 | 0,45 | 8,99E-03 |
| MGI:109495 | Nfil3 | nuclear factor, interleukin 3, regulated | 0,45 | 4,73E-03 |
| MGI:1915420 | B230118H07Rik | RIKEN cDNA B230118H07 gene | 0,45 | 8,42E-03 |
| MGI:96974 | Kitl | kit ligand | 0,45 | 4,56E-03 |
| MGI:1923173 | Dnttip2 | deoxynucleotidyltransferase, terminal, interacting protein 2 | 0,45 | 1,10E-02 |
| MGI:2445092 | Utp14b | UTP14, U3 small nucleolar ribonucleoprotein, homolog B | 0,45 | 1,31E-02 |
| MGI:1919922 | Zfp518a | zinc finger protein 518A | 0,44 | 6,55E-03 |
| MGI:108187 | Zfp160 | zinc finger protein 160 | 0,44 | 8,90E-03 |
| MGI:1923385 | Prex2 | phosphatidylinositol-3,4,5-trisphosphate-dependent Rac exchange factor 2 | 0,44 | 3,12E-03 |
| MGI:1333774 | Mrpl42 | mitochondrial ribosomal protein L42 | 0,44 | 5,05E-03 |
| MGI:104754 | Sik1 | salt inducible kinase 1 | 0,44 | 4,56E-03 |
| MGI:88495 | Crem | cAMP responsive element modulator | 0,44 | 7,46E-03 |
| MGI:1913368 | Sarnp | SAP domain containing ribonucleoprotein | 0,44 | 1,09E-02 |
| MGI:2389572 | Brcc3 | BRCA1/BRCA2-containing complex, subunit 3 | 0,44 | 6,61E-03 |
| MGI:2444487 | Zbtb41 | zinc finger and BTB domain containing 41 homolog | 0,44 | 4,49E-03 |
| MGI:3036250 | A530054K11Rik | RIKEN cDNA A530054K11 gene | 0,44 | 1,71E-02 |
| MGI:2442829 | Klhl4 | kelch-like 4 | 0,44 | 1,18E-02 |
| MGI:1261910 | Pibf1 | progesterone immunomodulatory binding factor 1 | 0,44 | 1,58E-02 |
| MGI:1918101 | Tbc1d8b | TBC1 domain family, member 8B | 0,44 | 1,88E-02 |
| MGI:1914269 | Actr6 | ARP6 actin-related protein 6 | 0,43 | 4,56E-03 |
| MGI:1918180 | Nol8 | nucleolar protein 8 | 0,43 | 1,44E-02 |
| MGI:2149842 | Sdf2l1 | stromal cell-derived factor 2-like 1 | 0,43 | 3,74E-03 |
| MGI:105053 | Hsph1 | heat shock 105kDa/110kDa protein 1 | 0,43 | 3,97E-04 |
| MGI:1922866 | Smim15 | small integral membrane protein 15 | 0,43 | 1,05E-02 |
| MGI:1913830 | Esf1 | ESF1, nucleolar pre-rRNA processing protein, homolog | 0,43 | 1,68E-02 |
| MGI:1926209 | Dek | DEK oncogene (DNA binding) | 0,43 | 9,55E-03 |
| MGI:1355295 | Hmgn5 | high-mobility group nucleosome binding domain 5 | 0,43 | 2,25E-02 |
| MGI:109153 | Ktn1 | kinectin 1 | 0,43 | 4,71E-03 |
| MGI:1344403 | Cfdp1 | craniofacial development protein 1 | 0,43 | 4,41E-03 |
| MGI:1933972 | Tra2a | transformer 2 alpha homolog | 0,42 | 3,65E-04 |
| MGI:1927339 | Mpp5 | membrane protein, palmitoylated 5 (MAGUK p55 subfamily member 5) | 0,42 | 1,13E-02 |
| MGI:3621440 | Zfp938 | zinc finger protein 938 | 0,42 | 1,88E-02 |
| MGI:1930708 | Zfp386 | zinc finger protein 386 (Kruppel-like) | 0,42 | 1,25E-02 |
| MGI:2675306 | Zfp930 | zinc finger protein 930 | 0,42 | 2,80E-02 |
| MGI:1352456 | Nr4a2 | nuclear receptor subfamily 4, group A, member 2 | 0,42 | 3,65E-04 |
| MGI:1916455 | Zfp748 | zinc finger protein 748 | 0,41 | 2,34E-02 |
| MGI:3650228 | Gm13341 | predicted gene 13341 | 0,41 | 1,93E-02 |
| MGI:104860 | Fxr1 | fragile X mental retardation gene 1, autosomal homolog | 0,41 | 4,29E-03 |
| MGI:1306780 | Egr3 | early growth response 3 | 0,41 | 6,98E-03 |
| MGI:3664583 | Cntnap5b | contactin associated protein-like 5B | 0,41 | 2,36E-02 |
| MGI:2660877 | Gucy1a2 | guanylate cyclase 1, soluble, alpha 2 | 0,41 | 1,36E-02 |
| MGI:2135609 | Lin7a | lin-7 homolog A | 0,41 | 7,02E-03 |
| MGI:1919747 | Zc3h15 | zinc finger CCCH-type containing 15 | 0,41 | 1,31E-02 |
| MGI:3040706 | Zfp738 | zinc finger protein 738 | 0,41 | 1,40E-02 |
| MGI:1917613 | Fam135b | family with sequence similarity 135, member B | 0,41 | 1,10E-02 |
| MGI:2146320 | Dock10 | dedicator of cytokinesis 10 | 0,41 | 4,29E-03 |
| MGI:99185 | Zfp40 | zinc finger protein 40 | 0,41 | 1,79E-02 |
| MGI:106184 | Npm1 | nucleophosmin 1 | 0,41 | 1,21E-02 |
| MGI:95301 | Eif3a | eukaryotic translation initiation factor 3, subunit A | 0,40 | 2,00E-03 |
| MGI:1913500 | Taf13 | TAF13 RNA polymerase II, TATA box binding protein (TBP)-associated factor | 0,40 | 7,22E-03 |
| MGI:95821 | Grin2b | glutamate receptor, ionotropic, NMDA2B (epsilon 2) | 0,40 | 2,32E-03 |
| MGI:88516 | Cryab | crystallin, alpha B | 0,40 | 1,11E-03 |
| MGI:1859646 | Golga4 | golgi autoantigen, golgin subfamily a, 4 | 0,40 | 6,55E-03 |
| MGI:1922869 | Fastkd2 | FAST kinase domains 2 | 0,40 | 1,10E-02 |
| MGI:1099792 | Ptpn4 | protein tyrosine phosphatase, non-receptor type 4 | 0,40 | 1,10E-02 |
| MGI:1922665 | Arhgap12 | Rho GTPase activating protein 12 | 0,40 | 7,22E-03 |
| MGI:1890752 | Zfp81 | zinc finger protein 81 | 0,40 | 1,10E-02 |
| MGI:97763 | Prlr | prolactin receptor | 0,40 | 2,75E-02 |
| MGI:2444631 | 4932438A13Rik | RIKEN cDNA 4932438A13 gene | 0,40 | 1,31E-02 |
| MGI:2442380 | 9330132A10Rik | RIKEN cDNA 9330132A10 gene | 0,39 | 1,71E-02 |
| MGI:2449975 | Iqgap2 | IQ motif containing GTPase activating protein 2 | 0,39 | 1,28E-02 |
| MGI:95620 | Gabrb2 | gamma-aminobutyric acid (GABA) A receptor, subunit beta 2 | 0,39 | 7,86E-03 |
| MGI:1920701 | Zfp763 | zinc finger protein 763 | 0,39 | 2,33E-02 |
| MGI:97286 | Ncl | nucleolin | 0,39 | 7,09E-03 |
| MGI:1196437 | Hltf | helicase-like transcription factor | 0,39 | 1,44E-02 |
| MGI:1917870 | Ube2v2 | ubiquitin-conjugating enzyme E2 variant 2 | 0,39 | 2,04E-02 |
| MGI:892979 | Chm | choroidermia | 0,39 | 1,44E-02 |
| MGI:894681 | Usp9x | ubiquitin specific peptidase 9, X chromosome | 0,39 | 1,24E-02 |
| MGI:106018 | St8sia4 | ST8 alpha-N-acetyl-neuraminide alpha-2,8-sialyltransferase 4 | 0,39 | 2,94E-02 |
| MGI:2144474 | Smek2 | SMEK homolog 2, suppressor of mek1 | 0,39 | 1,79E-02 |
| MGI:1913975 | Lrrk2 | leucine-rich repeat kinase 2 | 0,39 | 9,62E-03 |
| MGI:97623 | Plp1 | proteolipid protein (myelin) 1 | 0,39 | 4,29E-03 |
| MGI:2446700 | St18 | suppression of tumorigenicity 18 | 0,39 | 3,07E-02 |
| MGI:1914944 | Ift74 | intraflagellar transport 74 | 0,39 | 2,82E-02 |
| MGI:1919905 | Snhg5 | small nucleolar RNA host gene 5 | 0,39 | 1,79E-02 |
| MGI:1929510 | Zfand6 | zinc finger, AN1-type domain 6 | 0,39 | 7,09E-03 |
| MGI:99662 | Zfp62 | zinc finger protein 62 | 0,39 | 2,77E-02 |
| MGI:1196261 | Trmt10c | tRNA methyltransferase 10C | 0,38 | 2,23E-02 |
| MGI:2144305 | Ccdc55 | coiled-coil domain containing 55 | 0,38 | 2,69E-02 |
| MGI:101916 | Fabp7 | fatty acid binding protein 7, brain | 0,38 | 3,55E-02 |
| MGI:3040702 | Zfp874b | zinc finger protein 874b | 0,38 | 1,10E-02 |
| MGI:1921488 | Mterf2 | mitochondrial transcription termination factor 2 | 0,38 | 3,38E-02 |
| MGI:1916145 | Rasl11a | RAS-like, family 11, member A | 0,38 | 2,33E-02 |
| MGI:1913976 | Nipbl | Nipped-B homolog | 0,38 | 1,10E-02 |
| MGI:1353460 | Fkbp3 | FK506 binding protein 3 | 0,38 | 1,18E-02 |
| MGI:2445214 | Lrif1 | ligand dependent nuclear receptor interacting factor 1 | 0,38 | 2,10E-02 |
| MGI:2679257 | Zfp760 | zinc finger protein 760 | 0,38 | 4,69E-02 |
| MGI:1913493 | C1galt1c1 | C1GALT1-specific chaperone 1 | 0,38 | 7,20E-03 |
| MGI:104807 | Pls3 | plastin 3 (T-isoform) | 0,38 | 1,06E-02 |
| MGI:2683212 | Ranbp6 | RAN binding protein 6 | 0,38 | 2,35E-02 |
| MGI:1338833 | Dio2 | deiodinase, iodothyronine, type II | 0,38 | 1,89E-02 |
| MGI:2685921 | Apold1 | apolipoprotein L domain containing 1 | 0,38 | 4,34E-02 |
| MGI:2448380 | Hist1h2be | histone cluster 1, H2be | 0,38 | 4,69E-02 |
| MGI:95773 | Gnai3 | guanine nucleotide binding protein (G protein), alpha inhibiting 3 | 0,38 | 1,68E-02 |
| MGI:1921920 | Zfp943 | zinc finger prtoein 943 | 0,38 | 3,49E-02 |
| MGI:107735 | Myo9a | myosin IXa | 0,38 | 2,23E-02 |
| MGI:892001 | Slc22a6 | solute carrier family 22 (organic anion transporter), member 6 | -0,63 | 1,72E-04 |
| MGI:106012 | Tagln | transgelin | -0,55 | 1,87E-04 |
| MGI:98287 | Srsf5 | serine/arginine-rich splicing factor 5 | -0,54 | 3,75E-05 |
| MGI:99261 | Ptgds | prostaglandin D2 synthase (brain) | -0,54 | 9,64E-07 |
| MGI:1914723 | Slc47a1 | solute carrier family 47, member 1 | -0,51 | 5,18E-03 |
| MGI:88491 | Crabp2 | cellular retinoic acid binding protein II | -0,51 | 4,13E-03 |
| MGI:2143217 | Slc6a20a | solute carrier family 6 (neurotransmitter transporter), member 20A | -0,49 | 1,83E-03 |
| MGI:894696 | Serping1 | serine (or cysteine) peptidase inhibitor, clade G, member 1 | -0,49 | 5,13E-03 |
| MGI:88468 | Col1a2 | collagen, type I, alpha 2 | -0,47 | 6,61E-03 |
| MGI:88019 | Amy1 | amylase 1, salivary | -0,47 | 2,77E-03 |
| MGI:1197012 | Aebp1 | AE binding protein 1 | -0,47 | 3,88E-03 |
| MGI:98738 | Thbs2 | thrombospondin 2 | -0,45 | 4,56E-03 |
| MGI:2147913 | Igsf1 | immunoglobulin superfamily, member 1 | -0,45 | 1,83E-03 |
| MGI:88246 | Anxa2 | annexin A2 | -0,45 | 2,58E-03 |
| MGI:87963 | Agt | angiotensinogen (serpin peptidase inhibitor, clade A, member 8) | -0,44 | 1,40E-03 |
| MGI:1929938 | Perp | PERP, TP53 apoptosis effector | -0,44 | 6,61E-03 |
| MGI:2444654 | Ranbp3l | RAN binding protein 3-like | -0,43 | 1,96E-02 |
| MGI:95895 | H2-Aa | histocompatibility 2, class II antigen A, alpha | -0,43 | 2,23E-02 |
| MGI:1349645 | Islr | immunoglobulin superfamily containing leucine-rich repeat | -0,43 | 6,98E-03 |
| MGI:96976 | Mgp | matrix Gla protein | -0,42 | 4,33E-04 |
| MGI:1351330 | Cartpt | CART prepropeptide | -0,42 | 1,64E-03 |
| MGI:893588 | Cirbp | cold inducible RNA binding protein | -0,42 | 5,18E-03 |
| MGI:1309511 | Cdc7 | cell division cycle 7 | -0,41 | 1,38E-02 |
| MGI:2651811 | Tph2 | tryptophan hydroxylase 2 | -0,41 | 3,07E-02 |
| MGI:107928 | Aldh1a2 | aldehyde dehydrogenase family 1, subfamily A2 | -0,40 | 2,69E-02 |
| MGI:2442367 | Slc13a4 | solute carrier family 13 (sodium/sulfate symporters), member 4 | -0,40 | 2,40E-02 |
| MGI:97631 | Pmp22 | peripheral myelin protein 22 | -0,40 | 3,02E-04 |
| MGI:88460 | Col6a2 | collagen, type VI, alpha 2 | -0,40 | 7,09E-03 |
| MGI:103017 | Fcgrt | Fc receptor, IgG, alpha chain transporter | -0,39 | 6,61E-03 |
| MGI:95629 | Slc6a13 | solute carrier family 6 (neurotransmitter transporter, GABA), member 13 | -0,39 | 1,28E-02 |
| MGI:1914856 | Fibin | fin bud initiation factor homolog | -0,39 | 2,35E-02 |
| MGI:2441845 | Snhg11 | small nucleolar RNA host gene 11 | -0,38 | 4,29E-03 |
| MGI:1202864 | Myoc | myocilin | -0,38 | 1,26E-02 |
| MGI:2149635 | Slc13a3 | solute carrier family 13 (sodium-dependent dicarboxylate transporter), member 3 | -0,38 | 5,22E-03 |

**Supplemental Table 1: Up- or down-regulated genes in WT-EE compared to WT-SH mice**
